# Molecular evolutionary mechanisms driving functional diversification of α-glucosidase in Lepidoptera

**DOI:** 10.1038/srep45787

**Published:** 2017-04-12

**Authors:** Xiaotong Li, Liangen Shi, Yanyan Zhou, Hongqing Xie, Xiangping Dai, Rongqiao Li, Yuyin Chen, Huabing Wang

**Affiliations:** 1 College of Animal Sciences, Zhejiang University, Hangzhou 310058, China

## Abstract

The digestive tract of lepidopteran insects is unique given its highly alkaline pH. The adaptive plasticity of digestive enzymes in this environment is crucial to the highly-efficient nutritional absorption in Lepidoptera. However, little is known about the molecular adaptation of digestive enzymes to this environment. Here, we show that lepidopteran α-glucosidase, a pivotal digestive enzyme, diverged into sucrose hydrolase (SUH) and other maltase subfamilies. SUH, which is specific for sucrose, was only detected in Lepidoptera. It suggests that lepidopteran insects have evolved an enhanced ability to hydrolyse sucrose, their major energy source. Gene duplications and exon-shuffling produced multiple copies of *α-glucosidase* in different microsyntenic regions. Furthermore, SUH showed significant functional divergence (FD) compared with maltase, which was affected by positive selection at specific lineages and codons. Nine sites, which were involved in both FD and positive selection, were located around the ligand-binding groove of SUH. These sites could be responsible for the ligand-binding preference and hydrolytic specificity of SUH for sucrose, and contribute to its conformational stability. Overall, our study demonstrated that positive selection is an important evolutionary force for the adaptive diversification of α-glucosidase, and for the exclusive presence of membrane-associated SUHs in the unique lepidopteran digestive tract.

The Lepidoptera (butterflies and moths) is one of the most widespread and widely recognisable insect orders in the world. This order contains approximately 180,000 described species in 126 families and 46 superfamilies^1^. The larvae of many lepidopteran species are major pests and are considered to be the most economically damaging pests in agriculture. The digestive system of Lepidoptera is quite different from that of other insects and is more complex^2^. All the digestive enzymes of Lepidoptera, other than those for initial digestion, are immobilised at the surface of the midgut cells^2^. In addition, the digestive tract of Lepidoptera is unique because of its extremely alkaline pH^3^,^4^, and the pH values measured in particular compartments of the larval digestive tract span a range between 9 and 11^2^,^5^. The lepidopteran gut is highly alkaline due to specific dietary preferences^6^,^7^,^8^, such as feeding on tannin-rich leaves^9^. The digestive enzymes, which have evolved into a specific pH optimum, should match the midgut condition for maximum efficiency. However, the molecular mechanisms of the phylogeny and adaptation of lepidopteran digestive enzymes are still poorly understood.

Sucrose is one of the main products of photosynthesis and the most common transported sugar in plants, and it is also an easily assimilated macronutrient that provides a carbon or energy source for insects. Insect sucrases catalyse the hydrolysis of sucrose into its constituent monosaccharides, which can be used by insects as a food source. Insect sucrase activity is generally thought to depend mainly on α-glucosidase (EC 3.2.1.20). However, sucrose hydrolases in the larval midgut of Lepidoptera have three distinct forms: an α-glucosidase, also known as maltase; a β-fructofuranosidase, which is acquired via horizontal gene transfer (HGT) from bacteria; and a sucrose hydrolase (SUH), which displays specificity for sucrose^10^,^11^,^12^,^13^. Unlike typical α-glucosidase and β-fructofuranosidase, the SUH, which is associated with the midgut membrane, displayed measurable activity only against sucrose and showed a very broad range of pH optima, ranging from approximately pH 6 up to 11. Recently, sucrose hydrolases categorised into α-glucosidases were found in three lepidopteran species, *Bombyx mori, Trilocha varians* and *Samia cynthia ricini*, and were named as BmSUH, TvSUH and ScSUH, respectively^14^. Recent genome sequencing projects have shown that SUH sequences are present in several lepidopteran species and absent from other insect orders. Although SUHs belong to α-glucosidases^15^, the SUHs were clearly distinct from other α-glucosidases, suggesting that SUHs have diverged from other α-glucosidases during the evolution of lepidopteran insects. Therefore, the evolution of α-glucosidases in Lepidoptera is highly unusual.

Insect α-glucosidases have been studied extensively in Brachycera and Nematocera, and are likely results of an ancient series of duplications^15^,^16^,^17^. α-glucosidase family underwent a complicated evolutionary history in Diptera, and some amino acid sites might be subjected to the pressure of positive selection or force of functional divergence (FD), which is expected to result in diversified functions. Although a large variety of *α-glucosidase* genes have been recently identified in Diptera^15^,^16^,^17^,^18^, only a few lepidopteran *α-glucosidases* were characterised, and not much is known about how such essential enzymatic functions evolve and diversify^14^. The recent sequencing of lepidopteran genomes has made the exploration of the molecular functions and evolutionary adaptations of SUH and other α-glucosidases in Lepidoptera possible.

In this study, we provide the first comprehensive analysis of the evolution of the α-glucosidase family in Lepidoptera. We found that α-glucosidase could be divided into two subfamilies, SUH and lepidopteran maltase (LMal), approximately 232 MYA in seven lepidopteran species. SUH and LMal were duplicated to several homologues subsequently, and the splice sites were conserved in each subfamily but differ from each other. FD and selection pressure analyses indicated that SUH or LMal fall under the effects of FD and positive selection, and we detected some amino acid residues that are prone to being influenced. In addition, we found that nine sites, which were involved in both FD and positive selection, were located around the ligand-binding groove of SUH. This study provides an example of how distinct selective pressure, along with ancestral gene differentiation and subsequent gene duplications, can contribute to lineage-specific adaptations and the divergence of lepidopteran digestive enzymes.

## Results

### Phylogenetic relationship of LMal and SUH in Lepidoptera

**Figure 1:**
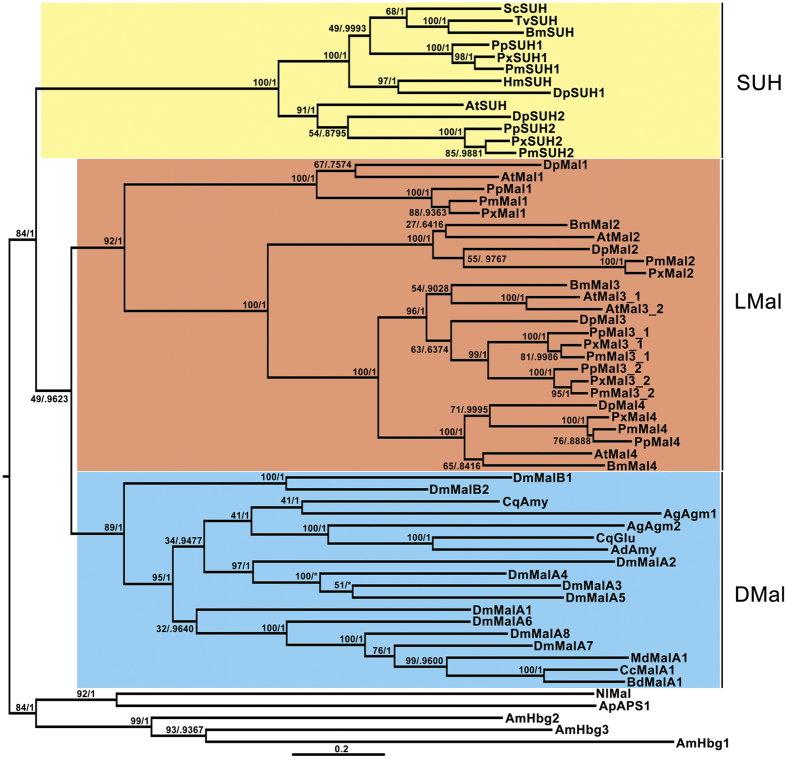
Reconstruction of the phylogeny of insect α-glucosidases. The ML tree depicts the evolutionary relationships among 62 sequences from species representing distinct insect lineages. Statistical supports corresponding to ML LRT and BA posterior probability are shown next to the corresponding nodes at relevant clades. Branch lengths in the tree are proportional to evolutionary distances between nodes, with the scale bar indicating the number of inferred amino acid substitutions per site. SUH, LMal and DMal are short for sucrose hydrolases, lepidopteran maltase, dipteran maltase, respectively.

In this study, we confirmed the evolutionary relationship between the maltase of Lepidoptera and those of other insect orders. We also determined whether SUH emerged before or after the divergence of LMal and other insect maltases. We retrieved and aligned 62 sequences of the insect α-glucosidase family from the NCBI and Ensembl databases using DmMal1A and BmSUH as query sequences (Supplementary Table S1). After reconstructing the phylogenetic tree by maximum likelihood (ML) and Bayesian methods, we found that the phylogenetic relationship of the insects α-glucosidases was well defined. Each subfamily represented a distinctive monophyletic clade, as supported by the high confidence values observed (Fig. 1). SUH, a subfamily of maltase from Lepidoptera, split from the maltase clade of lepidopteran and dipteran insects (Fig. 1). *LMal* was duplicated at least thrice to form a large gene family. By contrast, the *SUH* subfamily duplicated into the *SUH1* and *SUH2* clusters. Moreover, SUH and LMal shared conserved structural characteristics that harboured an α-amylase catalytic (α-amy) domain and seven conserved sequence regions (CSRs) (Supplementary Fig. S1). In addition, SUH possessed an N-terminus hydrophobic amino acid sequence (except AtSUH) (Supplementary Fig. S1). Recent studies have proved that the genes in this distinct clade, specifically, *BmSUH, TvSUH* and *ScSUH*, encode membrane-associated sucrose-specific hydrolases at the molecular level^14^.

**Figure 2:**
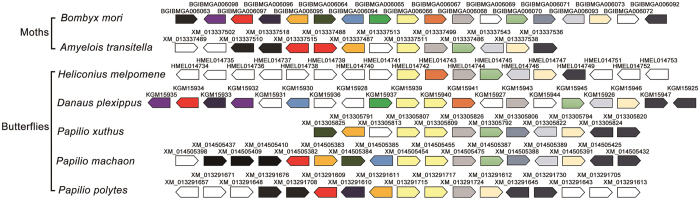
Conserved syntenic analysis of *SUH* and its surrounding genes. Using silkworm *BmSUH* as an anchor site, homologues of genomic genes linked to *SUH* were founded in six other lepidopteran chromosomes. *SUHs* of Lepidoptera are placed in the middle and colored in yellow. Homologues are represented by the same color, and genes with no homologue were indicated by blank boxes. The direction of genes is represented by block arrows. The positions of genes on chromosomes are not drawn to scale.

To ascertain the genomic relationship of *SUH1* and *SUH2*, we performed syntenic analyses around the region of *SUH1* and *SUH2* on the chromosomes of seven lepidopteran species (Fig. 2). Only one copy of *SUH* was detected in the genome of *B. mori, H. melpomene* and *A. transitella*. However, the *SUH* gene was duplicated to double adjacent copies, *SUH1* and *SUH2*, in *D. plexippus, P. xuthus, P. machaon* and *P. polytes*. Conserved gene organisation up- and downstream of *SUH* was also found in seven lepidopteran species. RT-PCR shows that *PxSUH1* and *PxSUH2* are mainly expressed in the midgut of fifth-instar larvae of *P. xuthus*, and *PxSUH1* showed a more abundant expression compared with *PxSUH2* (Supplementary Fig. S2). This conserved syntenic relationship indicated that the *SUH* gene was conserved and emerged early during lepidopteran evolution, and its duplication likely occurred specifically among many Rhopalocera species, but not in moth.

### Evolutionary history and gene structure of LMal and SUH in Lepidoptera

**Figure 3:**
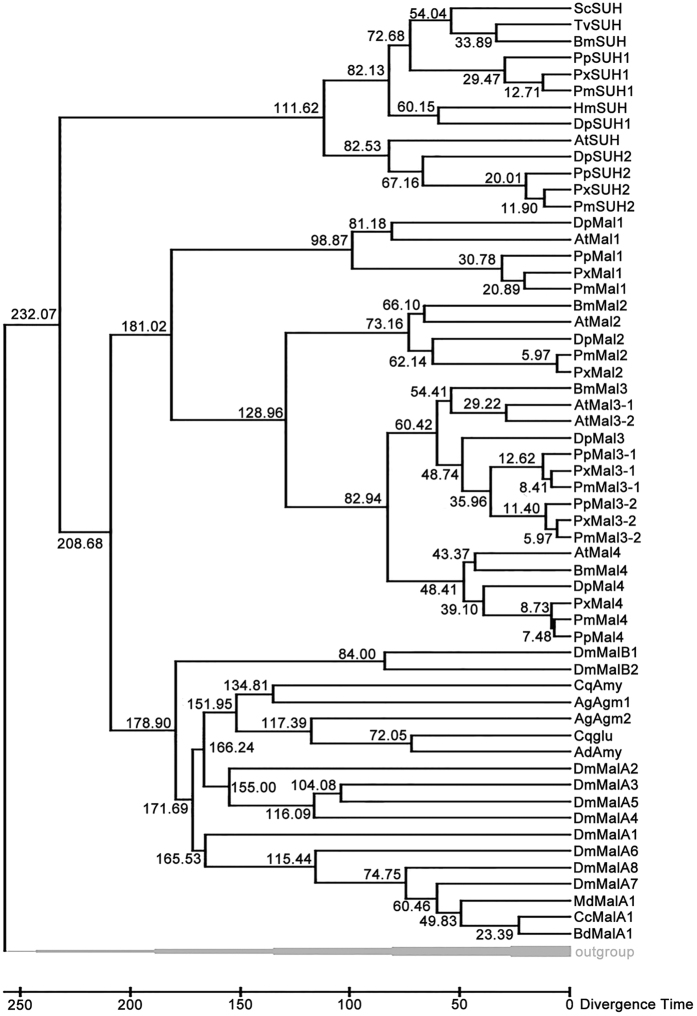
Time tree phylogenetic analysis of insect α-glucosidase family using the RelTime method. Numbers in the tree indicate the approximate relative times of divergence (MYA) between two lineages. Scale representation under the tree demonstrates divergence time of genes.

To shed light on the evolutionary history of *LMal* and *SUH*, we estimated the divergence time of particular gene duplications from the ML tree using RelTime method^19^,^20^ (Fig. 3). With two calibration points of divergence time between *DmMalB1* and *B2* (84 MYA) as well as *DmMalA2* and *DmMalA3/A4/A5* (155 MYA)^15^, we were convinced that the divergence time of *SUH/maltase* was approximately 232.07 MYA. After the differentiation of the common ancestral gene from which *SUH* and *maltase* are derived, *SUH* was divided into *SUH1* and *SUH2* approximately 111.62 MYA. Moreover, *LMal* and *DMal (maltase* of *Diptera*) were diverged approximately 208.68 MYA, and *LMal* subsequently was divided into *LMal1, LMal2, LMal3* and *LMal4* 98.87, 73.16, 60.42 and 48.41 MYA, respectively. Furthermore, the *LMal3* gene was further diverged into *LMal3-1* and *LMal3-2* in several lepidopteran species, and this duplication event occurred only 29.22–35.96 MYA. In addition, we conducted molecular dating by Bayesian inference methods with BEAST software (Supplementary Fig. S3). The BEAST estimation was similar with the divergence time calculated by RelTime method. Therefore, the evolutionary history analysis indicated that *SUH* and *LMal* were diverged from the last common ancestral gene a very long time ago, and some subsequent gene duplication episodes occurred specifically in certain lepidopteran species, which proved that the α-glucosidase family has experienced a complicated evolutionary process in Lepidoptera.

**Figure 4:**
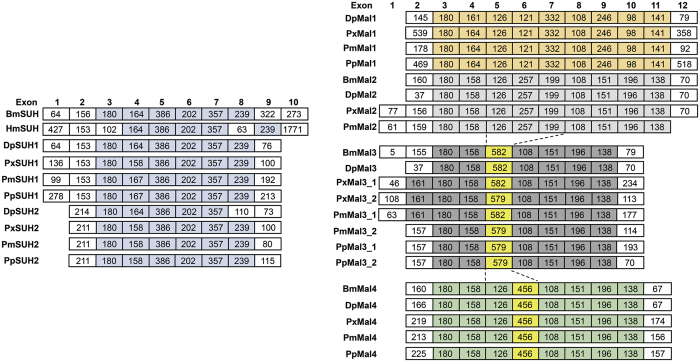
Exon/intron structures of *SUH* and *LMal* genes. The length of each exon is represented by the number in the box. Highly similar exon regions among each subfamily are indicated by the same color, and exons that may be generated by exon-shuffling are colored in yellow. Exon sizes are not drawn to scale.

Structural divergences are prevalent in duplicated genes and, in many cases, lead to the generation of functionally distinct paralogs^21^. Splice-site analysis was conducted to investigate the gene structures of *SUH* and *LMal* (Fig. 4). *SUH2* genes have minimal numbers of exons compared with *SUH1* and *LMal*, whereas the exons of *Mal1* are the largest. Moreover, the length of exons is conserved in *SUH* and *LMal*, and all genes harbour an exon with a length of 180 nt except *HmSUH*. Every subfamily of maltase has their own characteristics. *SUH* genes share linked exons of length 180-164 (or 158, 167)-386-202-357-239 nt except for *DpSUH2*, which lost the 239 nt exon. *LMal1* genes, which have the most conserved exon structure in maltase family, possess nine identical exons. Moreover, *BmMal2* and *DpMal2* share a very similar structure, and the length of each exon is identical except for the exon at the 5′ end. The splice sites of *PxMal2* and *PmMal2* are also analogous on their chromosomes, and eight exons share a consistent length. However, *LMal1* and *LMal2* were lost in the *B. mori* and *D. plexippus*, respectively. *LMal3* is duplicated in three *Papilio* species, and their structures are conserved after gene duplication, which contain linked-exons with lengths of 180-158-582 (579)-108-151-196-138 nt. *LMal4* genes possess a linkage region with eight exons, which is 180-158-126-456-108-151-196-138 nt in length.

Compared with *LMal1* and *LMal2*, the *LMal3* and *LMal4* homologues contain fewer exons, but they gain a longer exon with length of 582 (579) and 456 nt, respectively. We found that this larger exon could be generated by a duplication-induced exon-shuffling event. In *B. mori* and *D. plexippus*, the lengths of the fourth, fifth and sixth exons of *BmMal2/DpMal2* are 126, 257 and 199 nt, but the three exons were non-existent and a novel exon with a length of 582 nt appeared in *BmMal3/DpMal3*. Interestingly, the length of this larger exon was precisely equivalent to the sum of the fourth, fifth and sixth exons. A similar exon-shuffling event was also detected in two *Papilio* species, although the length of the larger exon (582 or 579 nt) of *Mal3* was not completely consistent with the sum of three small exons (585 nt). In *LMal4*, the fifth and sixth exons of *LMal2* were reconstructed into a larger exon of 456 nt. Moreover, *LMal2, LMal3* and *LMal4* are tandemly arranged in lepidopteran genomes, which suggest that *LMal3* and *LMal4* were generated by gene duplication of *LMal2*, and then exon-shuffling events occurred to form the contemporary gene structures. Splice-site analyses revealed that gene structure was conserved among the *SUH* subfamily but was not well conserved in the *LMal* subfamily, which underwent changes and exon-shuffling events.

### FD between SUH and maltase

Structural divergence occurred between SUH and LMal after gene duplications, and we wonder whether these two clusters underwent FD during evolution. To detect FD after gene duplications, we conducted analyses of type I functional divergence (FD I) between SUH and other maltases using Diverge 3.0^22^. By comparing SUH and maltase (LMal+DMal), SUH and Lmal, and SUH and DMal, the coefficients of FD I (θ) were 0.3232 ± 0.0577, 0.3935 ± 0.0630 and 0.2826 ± 0.0549, respectively (Table 1). This result indicated that functional constraint was altered significantly in SUH, and a remarkable FD between SUH and other maltases occurred. When comparing LMal versus DMal and SUH1 versus SUH2, the coefficients of FD I were clearly less, with values of 0.1980 ± 0.0516 and −0.4862 ± 0.023, respectively, which demonstrated that FD was weaker in these cases. Moreover, a total of 34, 30 and 25 critical amino acid sites, which likely are responsible for FD I, were also detected between SUH and LMal, SUH and DMal, and SUH and maltase, respectively. Most of the above-detected critical amino acid sites were located in the α-amy domain and these sites might be crucial to the changes in the enzyme properties and catalytic capability. The FD analyses indicated that the functions of SUH may be significantly changed in comparison with other maltases, which was consistent with the biochemical evidence that SUH is specific to sucrose hydrolysis but lost maltose digestion activity^14^.

### Detection of positive selection for SUH and LMal sequences

To understand the evolutionary basis of FD between SUH and LMal, we estimated the rates of nonsynonymous to synonymous nucleotide substitution (d_N_/d_S_ or ω) under different codon substitution-based evolutionary models. We employed likelihood ratio tests (LRT) with a site-specific model in the CodeML program of PAML4.8^23^. Under the most basic model M0 (assuming that ω is invariable among sites and branches), the value of ω was 0.04 among the whole maltase family, which indicated that most sites represent convincing purifying selection during maltase evolution. More realistic conditions allow ω to vary among sites following a β-distribution (models M7 and M8). M8 (β and ω > 1) model was a significantly better fit for the sequences in the ML tree, compared to the M7 (β) model (2ΔL = 20621.34, p < 0.001, Table 2). The value of ω was calculated as 2.06636 for the whole α-glucosidase family (Table 2). Most amino acid residues were under purifying selection, as a total of 70 sites, mainly in the α-amy domain, were identified as subject to positive selection under M8 using Bayes empirical Bayes (BEB) analysis with posterior probabilities ≥0.95 (Table 2)^24^,^25^. Among these sites, 251Y, a site that has been proven to determine the substrate specificity for maltose or sucrose in *A. mellifera*, was also detected under positive selection^26^.

To test whether certain lineages in SUH and LMal are under positive selection, a branch-specific model implemented in CodeML of PAML 4.8 was used to explore lineage-specific variation in selection pressure. The one-ratio model (H_0_) assumes a single ω for all lineages in the phylogenetic tree^27^. When we conducted free-ratio model (H_1_) analyses, which assumes a different ω parameter for each branch in the phylogenetic tree, H_1_ was found to fit significantly better to the data than did H_0_ (2ΔL = 499.04, p < 0.001, Table 2), suggesting that α-glucosidases are subjected to different selective pressures^27^. Positive selection was detected in the lineages of SUH (ω = 17.14), SUH2 (ω = 4.94), maltase (ω = 9.01), and ancestral lineage leading to LMal2, LMal3 and LMal4 (ω = 15.49) (Table 2), which reflected that positive selection occurred when gene duplications happened or when new members arose in α-glucosidase family.

Given that positive selection often affects a small subset of sites along particular lineages, the branch-site model was then used to detect positive selection in individual codons along SUH or maltase lineages, allowing ω to vary among both sites and branches^28^ (Table 2). Ratios of ω in different SUH and maltase lineages were varied, and lineages of SUH1, SUH2, LMal, LMal1, LMal3, LMal4 and ancestral lineages leading to SUH and LMal/DMal, LMal2 and LMal3/4, and LMal3 and LMal4 were significantly under positive selection. Moreover, some amino acid sites, which underwent positive selection, were also detected. We detected 16 and 4 sites subjected to significant positive selection from the entire SUH lineage (not significant) and SUH2 lineage, respectively. To the maltase of Lepidoptera, one, eight, one and six sites under positive selection were found in the lineages of LMal, LMal1, LMal2 and LMal4, respectively. In addition, another three or four sites were identified to bear positive selection when the divergence occurred between LMal2 and LMal3/4 or LMal3 and LMal4, respectively. Moreover, two additional sites were also detected to be under positive selection in the ancestral lineage, leading to SUH and maltase, and no site was detected under positive selection in the branches of SUH1 and LMal3. Among these sites, 191Q and 366Y were subjected to positive selection significantly both in the lineages of LMal1 and LMal4, whereas other 24 positive selection sites detected by the branch-site model had no commonality in each lineage. A summary of the above results is shown in Table 2. This result demonstrated that different lineages are subjected to various selective pressures, and positive selection sites, located in different parts of maltase, could contribute to the evolutionary diversity of each lineage, even resulting in ultimate functional differences.

### Protein structure modelling of SUH1

**Figure 5:**
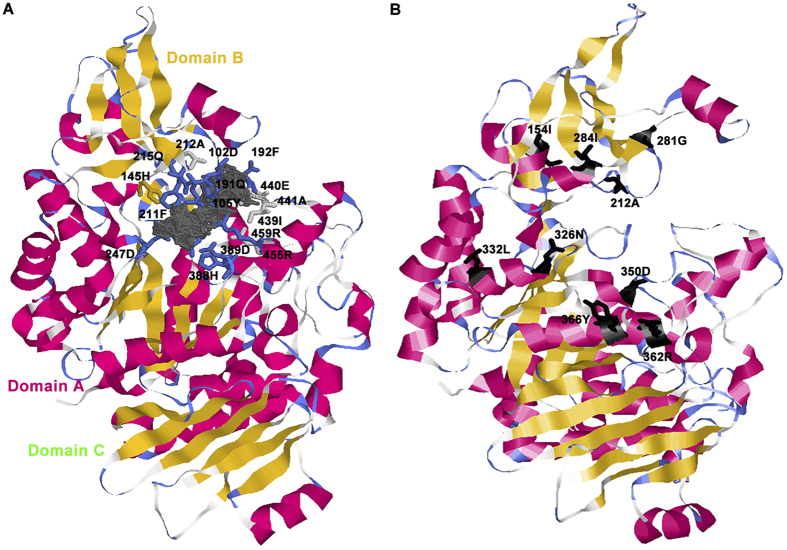
3D architecture of BmSUH showing positive selection and functional divergence residues. (**A**) Tertiary structure of BmSUH that binds to sucrose (complex colored in grey). Critical sites that predicted to be involved in ligand binding are mapped onto the structure and are represented as stick model. (**B**) Nine sites both contributed to positive selection and FD I are mapped onto the tertiary structure of BmSUH with black sticks. α-Helices, β-sheets and turns are shown in magenta, yellow and pale blue, respectively. All other residues are shown in white.

Although SUH1 showed significant homology to the maltase of insects, it exhibited substrate specificity for sucrose^14^. This functional diversity may depend upon the structural variation in the SUH1. To resolve the protein structure of SUH1, we built three-dimensional (3D) model by homology. The Phyre2 server was used to predict the tertiary structure of BmSUH with the intensive mode^29^(Fig. 5). By combining multiple template modelling and simplified *ab initio* folding simulation, we modelled the molecular structure of BmSUH, using the oligo-1,6-glucosidase (dextrin 6-α-glucanohydrolase, EC 3.2.1.10) from *Bacillus cereus* (PDB ID: 1uok) as the template. A total of 527 residues (87% of BmSUH) have been modelled with 100% confidence and 31% identity with the template. Approximately 31% and 16% of BmSUH is composed of α-helix and β-strand, respectively, whereas 3% of this protein is made up of transmembrane helix. Moreover, the 3D modelling showed that BmSUH contains three domains (Domains A, B and C), which are similar to other α-glucosidases (Fig. 5A).

The ligand-binding sites are important in determining the interaction between protein and its ligand, and the 3DLigandSite web server was used to predict potential binding sites^30^. A total of 16 amino acid sites were identified to be crucial for substrate binding (Fig. 5A). For the maltase family, an active site cleft usually exists between Domains A and B, and a triad of catalytic residues (Asp, Glu and Asp) are responsible for the catalytic reaction^31^. The result showed that 16 sites form a binding pocket to the substrate, and three catalytic residues are included in them. The 212A site is one of the potential binding sites. However, this site was also detected to be under positive selection by site-specific model that was responsible for the FD of SUH and maltase. Moreover, 191Q, another potential binding site, was also identified to be subjected to the positive force in LMal1 and LMal4 by branch-site model. These amino acid sites might be responsible for the functional differentiation and specific evolutionary adaptations during the evolution of α-glucosidase family in Lepidoptera. When the sites involved in both positive selection and FD were mapped to the structure of BmSUH, we found that these nine sites were mainly on the α-helix of the molecular surface, and were precisely located around the ligand-binding groove (Fig. 5B). This result indicated that specific ligand-binding sites would not be major targets for adaptive changes in the SUH family. However, the ordered distribution of sites, which involved both positive selection and FD, reflected that they could have effects on the discrepancy of ligand-binding and conformational stability.

## Discussion

The lepidopteran digestive system is characterised by two derived features, including developing extremely alkaline midguts and losing the midgut ceca^32^. These characters reflect the divergent selective pressure may have been imposed on the evolution of lepidopteran digestive system. In the present study, we found that the SUH subfamily of α-glucosidase is only detected in Lepidoptera, which showed high-alkaline adaptability. SUH and other LMal were diverged by ancestral gene expansion events, and their functions showed differentiation in subsequent evolutionary process. This differentiation may be caused by various selection pressures, which are exerted in different subfamilies. Adaptive selection pressure led to the exclusive presence of SUH1 in the highly alkaline digestive tract of Lepidoptera. Moreover, nine sites subjected to both positive selection and FD were located around the ligand-binding groove. The sites may contribute to the catalytic specificity to substrates and the stability of molecular conformation. The emergence of *SUH* and its subsequent duplications reflect effective adaptations to the specific diets and digestive environment of Lepidoptera^32^.

**Figure 6:**
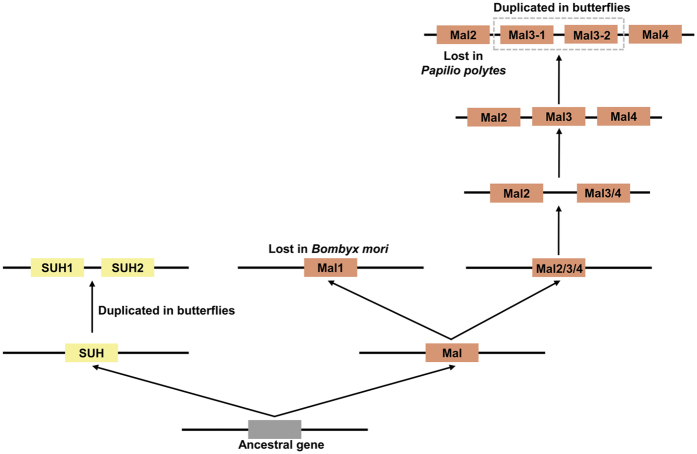
A proposed scenario of evolution and gene duplications of *α-glucosidases* in Lepidoptera. *SUH* and *LMal* were descended from the common ancestor, then went through genes gain and loss events in Lepidoptera. Members from *SUH* and *LMal* are distinguished by different colors.

The exon structures of *SUH* are conserved in Lepidoptera, but they significantly changed compared with *LMal* (Fig. 4). Unlike LMal, SUH possessed an N-terminal hydrophobic amino acid sequence (except AtSUH), which could potentially function as a membrane association region, explaining why SUHs are associated with membrane^14^. The gene structures appear to have great variety between *SUH* and *LMal* after the ancestral gene differentiation event ages ago, which may contribute to the functional diversification and differences in membrane-spanning domains and substrate specificity. The LMal subfamily, underwent a more complicated evolutionary process, with at least three rounds of gene duplication, whereas SUH was only duplicated once (Fig. 6). In addition, Mal1 is lost in *B. mori*, which may be an outcome of gene deletion or genome rearrangement. Recent works have started providing strong evidence for the functional diversification of α-glucosidase in Diptera and Hymenoptera, such as maltose hydrolysis (Agm1 and Agm2 of *A. gambiae*)^33^, sucrose degradation (HBG1 and HBG3 of *A. mellifera*), a receptor for Bin toxin (Cpm1 of *A. gambiae*)^34^, and heteromeric amino acid transporters (hcHATs proteins)^35^. Compared with Diptera species, the gene expansion of lepidopteran *α-glucosidase* is much simpler, as *Drosophila* experienced eight rounds of duplications and developed ten *α-glucosidase* genes^16^.

Synteny conservation analysis was performed to confirm the results of the phylogenetic analysis. *SUH* and its surrounding genes were tandemly arrayed in lepidopteran genomes (Fig. 2), but *SUH2* only emerged near *SUH* in several butterfly species^36^,^37^. Moreover, RT-PCR analysis showed a weaker expression of *PxSUH2* than *PxSUH1* in the midgut of *Papilio* species (Supplementary Fig. S2). *SUH2* may be generated by the duplication of *SUH1*, and this duplication event occurred in a few species not long ago. The emergence of *SUH2* reflects deep adaptation to the dietary habit or digestive needs of butterfly species, and further biochemical characterisation of *SUH2* will be of great interest. *LMal2, LMal3* and *LMal4* were also tandemly arrayed on the chromosome, but *LMal1* was located in separate chromosomal regions. This distribution model was similar to that of some Diptera species, as their *α-glucosidase* family is also located in two or three chromosomal regions^15^. In addition, gene structure analyses indicate that *LMal3* and *LMal4* were generated by exon-shuffling of *LMal2*. In this way to generate new genes was first observed during the evolution of *α-glucosidase* (Fig. 4). This result demonstrates that gene duplication and exon-shuffling contribute much to the *maltase* gene family expansion in Lepidoptera.

The subsequent divergence after gene duplication plays an important role in the evolution of novel gene function^38^. Many residues, including several functionally determined sites (212A, 251Y), were detected to be under positive selection, and key residues affected by diversified natural selection may result in the functional changes. 251Y/H has been previously found to be important in substrate preferences for sucrose or maltose^26^. This residue differs in SUH1 and SUH2, as SUH1 mainly harbours Y, whereas SUH2 harbours H. Interestingly, the residues corresponding to 251Y are in conserved sequence region II of the GH-13 enzyme. Region II has been noted as a determinant of the substrate specificity of GH-13 enzymes^26^. This result opens exciting avenues for future research where functional changes are caused by Y251H substitution in region II. In addition, strong signals of positive selection were detected during ancestral SUH divergence and in the SUH2 lineage, suggesting that the SUH subfamily has evolved an enhanced ability for sucrose digestion in response to the Lepidoptera-specific feeding habits and gastrointestinal circumstance. The evolution of the SUH subfamily was concordant with the theory that random mutations were fixed in one daughter gene under relaxed purifying selection, which occurred by the reduced functional constraint provided by genetic redundancy^39^,^40^. Compared with the ancestral gene, *SUH2* showed a weaker expression, and may undergo neofunctionalisation or subfunctionalisation during evolution. Moreover, four sites of SUH2, which were detected under positive selection by the branch-site model (Table 2), might contribute to the functional change. For LMal, positive selections had an effect on the leading branch of the whole LMal and ancestral branch of LMal2 and LMal3&4, but not on individual lineages of LMal immediately after gene divergence (Table 2). This result suggested that diversifying selection only acted upon the process of *LMal* gene divergence, but not on novel genes after duplication. Moreover, many positively selected sites in the core domain were detected from the whole α-glucosidase family of insect by the site-specific model, which indicated that the α-glucosidase family underwent a changeable evolutionary course. The α-glucosidase family should have been adaptively modified to recognise and bind different substrates and ensure the digestibility of varied diets.

If positive selection largely influenced the evolution of LMal and SUH, then how many changes occurred in the functions of these genes? To answer this question, we measured the FD I and critical sites involved in it by Diverge3 software, which demonstrated that altered functional constraints may occur after duplication, when SUH was compared with maltase, LMal or DMal. However, it suggested a functional constraint between SUH1 and SUH2 (Table 1). Critical amino acid residues, which may contribute to FD, were also detected, and all these sites were located in the α-amy domain when compared SUH with maltase (Table 1). Our results are consistent with previous studies, which have shown that BmSUH had substrate transformation to sucrose, unlike conventional maltases with maltose specificity^14^.

Although the structures of sucrose complexes with acid-base mutants of the GH13 enzymes have been examined, no 3D structures of the enzyme proteins in a complex with sucrose have yet been determined^26^. In this study, we predicted the tertiary structure and sucrose binding sites of BmSUH using Phyre2 and 3DLigandSite software^29^,^30^. Our predicted result was concordant with the estimation of Seddigh, who conducted homology modelling of α-glucosidase, such as Dm-NP610382 (*D. melanogaster*), Am-XP006560868 (*A. mellifera*), At-NP196733 (*A. thaliana*), Hs-NP937784 (*H. sapiens*) and Mt-YP007966392 (*M. tuberculosis*)^41^. This similarity of tertiary structure prediction analysis indicated that the 3D structures of α-glucosidase are conserved during evolution. Moreover, BmSUH harbours the potential 16 binding sites and forms a substrate-binding groove to bind and catalyse sucrose (Fig. 5A). When mapping nine sites, which were detected in both site-specific model analyses and FD analyses of SUH versus maltase, onto the modelled protein structure of BmSUH, we found that these sites were mainly located around the substrate-binding groove in the α-helices of Domain A (Fig. 5B). These sites might help to stabilise the protein conformation and assist ligand binding. Among the nine sites, the 212A site was identified to involve FD and positive selection, and also a site that is predicted to participate in the sucrose-binding reaction. Therefore, the nine sites, especially 212A, could be inferred as key sites during the evolution and functional formation of SUH, and they contributed to the recognition mechanism of substrate specificity. We propose these residues as targets for further experimental study of SUH functions. Daimon had proven that a β-fructofuranosidase (SUC), which is originally known as an ‘anomalous’ enzyme that had been believed to be absent in the animal kingdom, serves as a sucrose-digesting enzyme in the silkworm physiology^13^. Moreover, previous studies have shown that organisms, which access sucrose as a major food source, can acquire invertases from bacteria via horizontal gene transfer (HGT) to ensure the efficient utilisation of sucrose, such as plant-parasitic nematodes^42^. Recent genome sequencing projects have shown that SUC and SUH are present in lepidopteran insects, suggesting that Lepidoptera has evolved an enhance ability of digesting sucrose. The evolution of SUH, as a specific sucrose hydrolysis enzyme, reflects that lepidopteran insects can adapt to specific environments and diets by altering their original physiological characteristics.

## Materials and Methods

### Sequences collection and phylogenetic analyses

A comprehensive search by BLASTp and PSI-BLAST were performed in NCBI, Ensembl and FlyBase using DmMal1A and BmSUH as the query sequences^43^. After removing the partial sequences and redundant sequences, the final data set included 62 complete maltase and SUH sequences (Supplementary Table S1). All sequences were revised for errors in accession numbers and nomenclature. Multiple sequence alignments of these sequences were generated with MAFFT software^44^. According to the Akaike Information Criterion (AIC) for small sample size, MrModelTest2.3 revealed General Time Reversible model incorporating invariant sites and a gamma distribution (GTR+ I+ G) as the best model of molecular evolution with the best fit to our data^45^. Maximum-likelihood (ML) tree was reconstructed with RAxML-HPC BlackBox (8.2.8) on the CIPRES web portal (https://www.phylo.org/portal2) based on the GTR+ I+ G model^46^,^47^.

The Bayesian analyses were carried out using Markov chain Monte Carlo (MCMC) sampling in MrBayes3.2.1 with the same model described above, and data sets ran for 300,000 generations until they reached congruence^48^. The Bayesian tree was sampled every 100 generations, and the first 25% of the trees were discarded as burnin. Phylogenetic trees were visualized with FigTree 1.4.2.

### Estimation of evolutionary divergence times

To obtain temporal information on the divergence events, we implemented two methods to conduct molecular dating analyses. Frist, Reltime method of MEGA7 was used to infer the time tree by ML approach based on the GTR+ I+ G model. This method allows rates to vary from branch to branch without pre-specification of statistical distribution of lineage rates^19^,^20^.

Second, we estimated divergence times using Bayesian approach implemented in BEAST 1.83 with a relaxed molecular clock, which is determined by likelihood ratio test (LRT) of the molecular clock hypothesis (P < 0.01)^49^,^50^. Uncorrelated lognormal relaxed clock was chosen to estimate the evolutionary rate variations, and Yule speciation process was employed to model tree prior^51^. We set the number of generations to 10,000,000 with 10% burnin in MCMC analyses. Moreover, the maximum clade credibility (MCC) chronogram was summarized by TreeAnatator with posterior probability limit to 0.5. Two calibration constraints, divergence times of DmMal2- DmMal345 (84 MYA) and DmMalB1- DmMalB2 (155 MYA)^16^, were applied to date the divergence times of internal nodes within the phylogenetic tree. These analyses involved 62 nucleotide sequences described above.

### Expression analysis of *SUH* genes in *P. xuthus* by RT-PCR

Total RNA from the 3rd day of the fifth instar larvae of *P. xuthus* was used in the RT-PCR analysis. One microgram of total RNA was used to synthesize first-strand cDNA using PrimeScript RT reagent Kit with gDNA Eraser (Takara) according to the manufacturer’s instructions. The data were normalized by determination of the amount of gene encoding *ribosomal protein (rpl*) in each sample to eliminate variations in mRNA and cDNA quality and quantity. Gene-specific primers were deposited in Supplementary Table S2.

### Conserved synteny analyses

The syntenic relationship of *SUH* and its up- and downstream genes on lepidopteran genomes were revealed by the Genomics 30.01^52^ from Ensembl 31 database with *BmSUH* as the query gene. For genomes that not available on Ensembl, we searched genes around its corresponding orthologue of *BmSUH* from NCBI genome database manually^53^, and checked the result by reciprocal BLAST.

### Splice site and gene structure analyses

The Ensembl Metazoa genome browser release 31 and NCBI database were used to infer the exon boundaries of the coding regions of *SUH* and *LMal* genes. The accurate length (nt) of every exon was also determined.

### Analyses of type I functional divergence

Type I FD represents amino acid patterns that are highly conserved in one duplicate cluster but shows great variation in the other, which resulted in altered selective constraints between duplicated genes. The DIVERGE version 3.0 software was employed to test Type I FD after gene duplication^54^. The coefficient of FD (θ) is an indicator of the level of type I FD among two homologous gene clusters. The posterior probabilities (Q_k_) were also estimated to indicate amino acid sites to be responsible for FD. A value of Q_k_ > 0.7 was chosen as a cutoff to measure the degree of FD at the amino acid level, and Q_k_ > 0.9, which marked with an asterisk, was significant.

### Detection of positive selection

To measure the strength and mode of natural selection during the evolution of *SUH* and *LMal* gene subfamilies, the ratio of non-synonymous (d_N_) to synonymous substitutions (d_S_) (ω = d_N_/d_S_) was calculated by the CodeML program implemented in the PAML 4.8 package^23^. The phylogenetic tree was built by the ML method described above, and the alignment of sequences was achieved by MAFFT software. They were used to conduct CodeML analyses.

We employed three model, site-specific model, branch-specific model and branch-site model, to detect relative positive forces during the evolution of SUH and LMal. In the site-specific model, the M7 (β model) and M8 (β and ω > 1 model) were compared to identify the sites which under positive selection. The M7 model uses the flexible β distribution to indicate the difference of ω (value from 0 to 1) among different sites, whereas M8 allows several amino acid sites be under positive selection (ω > 1)^55^. If M8 fits the data of SUH and maltase better than M7, and detects sites under positive selection, we could accept the assumption of certain sites subjected to positive selection. In the branch-specific model, the significance of variation in the ω for each lineage was examined by free-ratio model^25^. If free-ratio model fits significantly better than the one-ratio model, which assigns a constant ω for all of branches in the tree, the alternative model could be accepted. Branch-site model, in which ω can vary in particular sites along particular branches, was used to test whether only several sites were under positive selection along foreground branch^28^. The null model, which caps ω = 1, was served to compare with the former model in the significance of fitness to the data. The likelihood ratio test (LRT) was used to test whether alternative model better fitted our data than null model significantly^25^,^56^.

### Protein structure and ligand-binding prediction

The Phyre2 structure prediction server (http://www.sbg.bio.ic.ac.uk/phyre2) was used to conduct tertiary structure prediction and alignment^29^. Phyre2 uses the alignment of hidden Markov models for homology-based protein modelling. It also incorporates the *ab initio* folding simulation to model regions with no detectable homology to known structures. Moreover, FD sites and positive selected sites were mapped to the predicted model. Predicted tertiary structures and relevant sites were visualized by RasMol 2.7.5^57^. The prediction of ligand-binding sites was employed with 3DLigandSite server, which based upon the modelled structure of query protein (http://www.sbg.bio.ic.ac.uk/3dligandsite)^30^.

## Additional Information

**How to cite this article:** Li, X. *et al*. Molecular evolutionary mechanisms driving functional diversification of α-glucosidase in Lepidoptera. *Sci. Rep.* **7**, 45787; doi: 10.1038/srep45787 (2017).

**Publisher's note:** Springer Nature remains neutral with regard to jurisdictional claims in published maps and institutional affiliations.

## Supplementary Material

Supplementary Information

## Figures and Tables

**Table 1:** Type I functional divergence (FD) of α-glucosidase family of insect.

FD	Subfamilies	Coefficient θ ± SE (P)	Critical Amino Acid sites
TypeI FD	SUH vs. LMal	0.3232 ± 0.0577 (P < 0.01)	101, 137, 164, **180**, **212**, 230, 232, 237, 250, 269, **279**, **281**, **284**, **288**, **292**, 296, 300, 307, 325, **326**, 329, 330, 331, **332**, 345, **350**, 360, **366**, 368, **369**, 397, 398, 415, 503
	SUH vs. DMal	0.3935 ± 0.0630 (P < 0.01)	141, 150, **154**, 161, 165, **191**, **212**, 222*, 237, **269**, **281**, 295, 329, 330, 332, 345, **350**, 360, 362*, 363, 364, 365, 366*, 370*, 373*, 374, 405, 415, 458, **468**
	LMal vs. DMal	0.1980 ± 0.0516 (P < 0.01)	148, 150*, 165, 229, 247, 265, 321, 362*, 365*, 369*, 370*, 373, 374, 398, 402, 454, 483
	SUH vs. Mal	0.2826 ± 0.0549 (P < 0.01)	101, **154,** 161, **212**, **222**, 237, 269, **281**, **284**, 295, 296, 325, **326**, 329, 330, 331, **332**, 345, 350*, 360, **362**, 364, **366**, 368, 373, 374, 415, 458
	SUH1 vs. SUH2	−0.4862 ± 0.023	287*, 380*, 393*, 496*

Functional divergences (Coefficient θ) for pairwise comparisons within the α-glucosidase family of insect are shown as value ± standard error. Critical amino acid sites detected as relating to FD with P > 70% (>90%, indicated with asterisks) are listed. Numbering refers to the positions in the alignments of protein sequences generated by MAFFT alignment. Residues that also under positive selection and relative to ligand-binding are presented by bold and underline, respectively.

**Table 2:** Tests of positive selection on Lepidopteran α-glucosidase family with site-specific, branch-specific and branch-site models.

Model	Foreground branch	-lnL	2lnL	P level	Parameter Estimates	Positive sites
**Site Model**						
M7		39876.24	20621.34	<0.01	p=0.67039, q=12.21042	not allowed
M8		50195.31			p0=0.99999, p=0.36811, q=1.82680 (p1=0.00001), ω=2.06636	97E**, 154I**, 158A**, 159R*, 180G**, 181V**, 202K**, 212A**, 213I**, 224A**, 234K**, 251Y**, 263R**, 272L**, 275F**, 277S**, 280L**, 281G**, 283T**, 284I**, 291L**, 309N**, 310K*, 311N**, 326N**, 327V**, 328S**, 332L**, 340A**, 341I**, 350D*, 355L*, 357S**, 358K**, 362R**, 366Y**, 367I**, 370R**, 377Y**, 379G**, 380I**, 391N*, 399H**, 400D**, 409N**, 411L**, 427R*, 429G, 466N**, 467S**, 468T**, 476T**, 477N**,484E**, 487Q**, 488E**, 489I**, 495K**, 496E**, 497T**, 499R**,507A**,510K**,513K**,585E**, 587T**, 588S**, 589S**, 590Q**, 591L**
**Branch-specific model**						
M0		41427.55	499.04	<0.01	ω=0.04	not allowed
Free-ratio model		41178.03			ωsuh=17.14, ωsuh1=0.10, ωsuh2=4.94, ωLMal1=0, ωLMal2=0.14, ωLMal3=0.18, ωLMal4=0.88, ωLMal=9.01, ωLMal234=15.49, ωLMal34=0.39	not allowed
**Branch-site model**						
Ma0	SUH1	41314.68	4.24	<0.05	ω0=0.04, ω1=1.00, ω2=1.00	not allowed
Ma		41312.56			ω0=0.04, ω1=1.00, ω2=999.00	none
Ma0	SUH2	41320.28	17.24	<0.01	ω0=0.04, ω1=1.00, ω2=1.00	not allowed
Ma		41311.66			ω0=0.04, ω1=1.00, ω2=21.51	184 P**, 195 S*, 222 N*, 288 T*
Ma0	Ancestral SUH	41305.76	0.96	>0.05	ω0=0.04, ω1=1.00, ω2=1.00	not allowed
Ma		41305.28			ω0=0.04, ω1=1.00, ω2=2.51	108S**, 150S**, 152Y**, 158A*, 181V*, 186S**, 191Q**, 279Q*, 280L*, 283T**, 292I**, 294L**, 357S*, 369L*, 407I**, 409N*
Ma0	Ancestral SUH and Mal	41313.43	13.67	<0.01	ω0=0.04, ω1=1.00, ω2=1.00	not allowed
Ma		41306.60			ω0=0.04, ω1=1.00, ω2=999.00	162G**, 483A*
Ma0	Ancestral LMal	41320.28	36.48	<0.01	ω0=0.04, ω1=1.00, ω2=1.00	not allowed
Ma		41302.04			ω0=0.04, ω1=1.00, ω2=999.00	199W*
Ma0	LMal1	41306.11	6.40	<0.05	ω0=0.04, ω1=1.00, ω2=1.00	not allowed
Ma		41302.91			ω0=0.04, ω1=1.00, ω2=10.07	181V*, 191Q*, 224A*, 226K*, 251Y**, 276E*, 366Y*, 588S*
Ma0	LMal2	41313.39	0.96	>0.05	ω0=0.04, ω1=1.00, ω2=1.00	not allowed
Ma		41312.91			ω0=0.04, ω1=1.00, ω2=2.14	341I*
Ma0	LMal3	41311.50	9.46	<0.01	ω0=0.04, ω1=1.00, ω2=1.00	not allowed
Ma		41306.77			ω0=0.04, ω1=1.00, ω2=999.00	None
Ma0	LMal4	41300.05	14.14	<0.01	ω0=0.04, ω1=1.00, ω2=1.00	not allowed
Ma		41292.98			ω0=0.04, ω1=1.00, ω2=999.00	147S**, 191Q*, 279Q*, 366Y*, 400D*, 585E*
Ma0	Ancestral LMal2, LMal3 and LMal4	41320.28	16.50	<0.01	ω0=0.04, ω1=1.00, ω2=1.00	not allowed
Ma		41312.03			ω0=0.04, ω1=1.00, ω2=119.75	161P*, 420Q*, 479S*
Ma0	Ancestral LMal3 LMal4	41320.05	9.56	<0.01	ω0=0.04, ω1=1.00, ω2=1.00	not allowed
Ma		41315.27			ω0=0.04, ω1=1.00, ω2=54.58	156S*, 203R**, 362R*, 371W**

The ω represents for d_N_/d_S_. * Significant at p< 0.05, ** Significant at p< 0.01. The site number was mapped to BmSUH after alignments. 2lnL, log-likelihood difference between compared models. Amino acid residues that also involved in FD I and ligand-binding were presented by bold and underline, respectively.
