# Supplementary material for: Molecular evolutionary mechanisms driving functional diversification of α-glucosidase in Lepidoptera
Source: Sci Rep. 2017 Apr 12;7:45787. doi: 10.1038/srep45787 (PMC5388851; doi:10.1038/srep45787)
Supplement: Supplementary Information [file srep45787-s1.pdf]

Supplementary Information

Molecular evolutionary mechanisms driving functional diversification of α-glucosidase in Lepidoptera

Xiaotong Li[1], Liangen Shi[1,*], Yanyan Zhou, Hongqing Xie, Xiangping Dai, Rongqiao Li, Yuyin Chen and Huabing Wang[*]

College of Animal Sciences, Zhejiang University, Hangzhou 310058, China

[1]: These authors contributed equally to this study.
[*]: Correspondence and requests for materials should be addressed to H.W. (wanghb@zju.edu.cn); L.S. (slgsilk@zju.edu.cn)

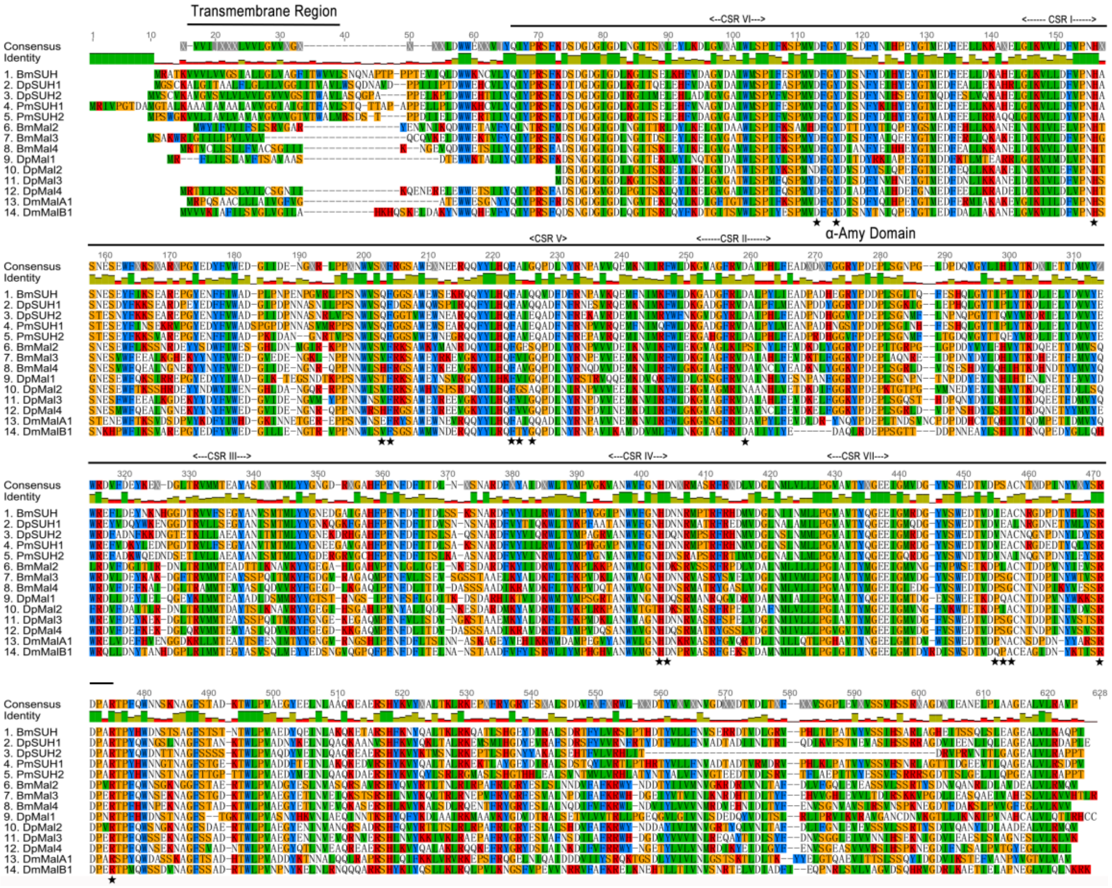

Figure S1. Amino acid sequence alignment of SUH and other maltase homologues. The lengths of two structural domains, the transmembrane region and α-amy domain, are indicated by black lines. Seven conserved sequence regions (CSRs) are also presented above the alignment blocks. Amino acid residues that are predicted to bind ligand are marked with pentagrams below the sites.

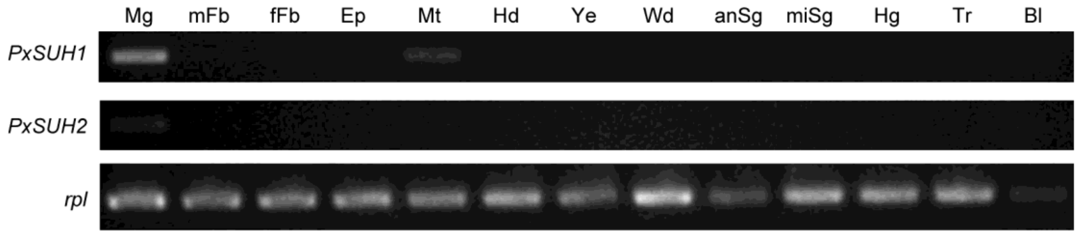

Figure S2. Expression profiles of *PxSUH1* and *PxSUH2*. Total RNA from the 3rd day of the fifth instar larvae of *P. xuthus* was used in the RT-PCR analysis. Tissues used for analysis were as follows. Mg, midgut; mFb, fat body (♂); fFb, fat body (♀) ; Ep, epidermis; Mt, Malpighian tubule; Hd, head; Ye, yellow; Wd, wing disc; anSg, anterior silk gland; miSg, middle part of the silk gland; Hg, hindgut; Tr, trachea; Bl, blood; and Ribosomal protein (rpl) was used as an internal control.

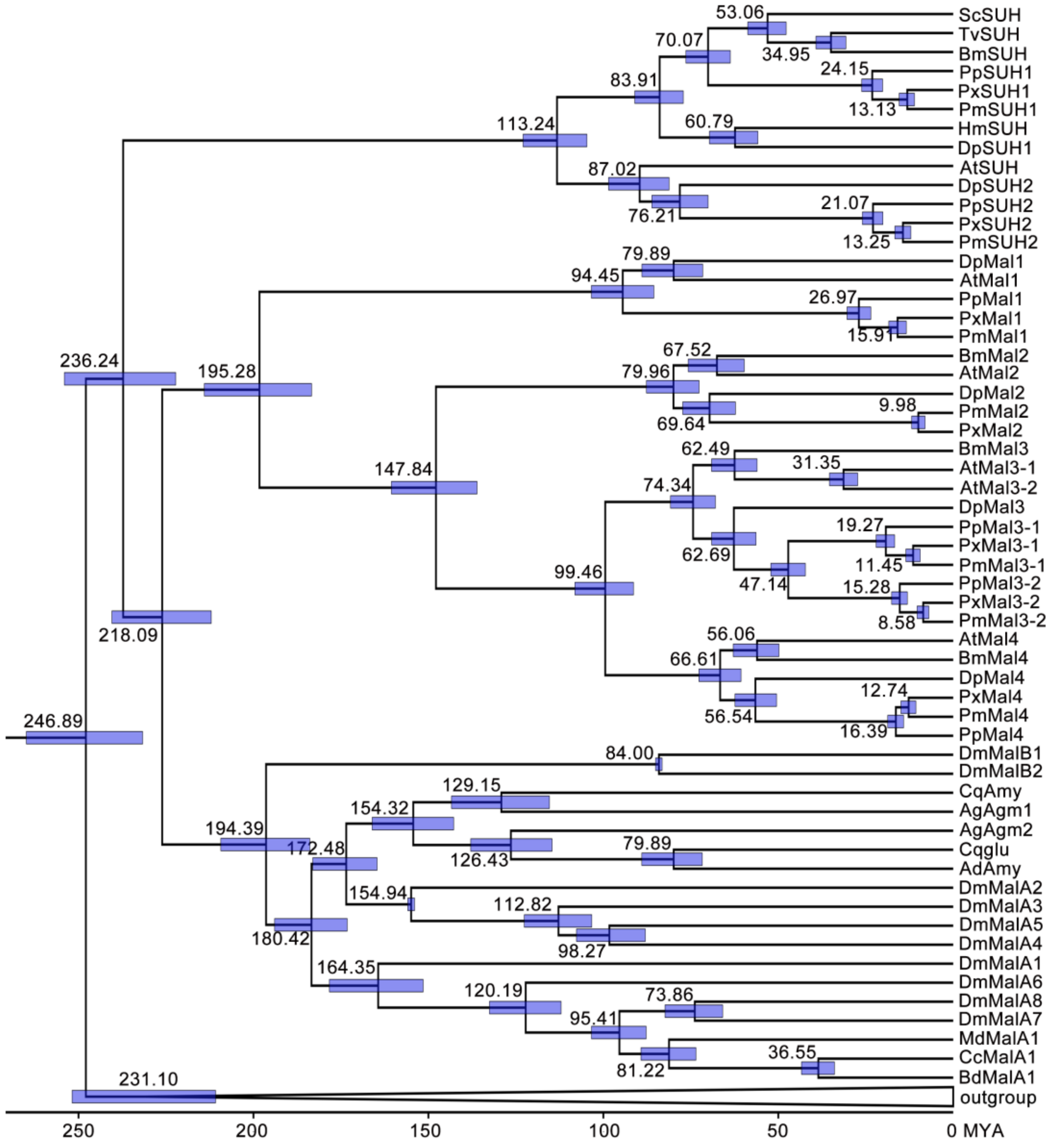

Figure S3. The maximum clade credibility (MCC) chronogram of α-glucosidase family inferred by BEAST 1.83. Numbers next to internal branches are the divergence times (MYA). Blue bars at internal nodes represent 95% higher posterior densities (95% HPD) of divergence time.

Table S1. GenBank accession numbers for sequences used in the phylogenetic analysis.

Name	Accession numbers	Species
**AtMal1**	XM_013345614	*Amyelois transitella*
**AtMal2**	XM_013345823	*Amyelois transitella*
**AtMal3_1**	XM_013345825	*Amyelois transitella*
**AtMal3_2**	XM_013345826	*Amyelois transitella*
**AtMal4**	XM_013345824	*Amyelois transitella*
**AtSUH**	XM_013337511	*Amyelois transitella*
**BmMal2**	XM_012694492	*Bombyx mori*
**BmMal3**	NM_001195462	*Bombyx mori*
**BmMal4**	XM_004930823	*Bombyx mori*
**BmSUH**	NM_001309622	*Bombyx mori*
**DpMal1**	EHJ72852	*Danaus plexippus*
**DpMal2**	EHJ72606	*Danaus plexippus*
**DpMal3**	EHJ72604	*Danaus plexippus*
**DpMal4**	EHJ72605	*Danaus plexippus*
**DpSUH1**	EHJ73356	*Danaus plexippus*
**DpSUH2**	EHJ73357	*Danaus plexippus*
**HmSUH**	HMEL014742	*Heliconius melpomene*
**PmMal1**	XM_014516262	*Papilio machaon*
**PmMal2**	XM_014513035	*Papilio machaon*
**PmMal3_1**	XM_014513031	*Papilio machaon*
**PmMal3_2**	XM_014513033	*Papilio machaon*
**PmMal4**	XM_014513032	*Papilio machaon*
**PmSUH1**	XM_014505454	*Papilio machaon*
**PmSUH2**	XM_014505455	*Papilio machaon*
**PpMal1**	XM_013281658	*Papilio polytes*
**PpMal3_1**	XM_013280617	*Papilio polytes*
**PpMal3_2**	XM_013280614	*Papilio polytes*
**PpMal4**	XM_013280615	*Papilio polytes*
**PpSUH1**	XM_013291715	*Papilio polytes*
**PpSUH2**	XM_013291717	*Papilio polytes*
**PxMal1**	XM_013322530	*Papilio xuthus*
**PxMal2**	XM_013312871	*Papilio xuthus*
**PxMal3_1**	XM_013312872	*Papilio xuthus*
**PxMal3_2**	XM_013312874	*Papilio xuthus*
**PxMal4**	XM_013312873	*Papilio xuthus*
**PxSUH1**	XM_013305807	*Papilio xuthus*
**PxSUH2**	XM_013305809	*Papilio xuthus*
**ScSUH**	BAP18685	*Samia ricini*
**TvSUH**	BAP18684	*Trilocha varians*
**DmMalA1**	AAF59089	*Drosophila melanogaster*
**DmMalA2**	AAF59088	*Drosophila melanogaster*
**DmMalA3**	AAM50308	*Drosophila melanogaster*

**DmMalA4**	ABY20547	*Drosophila melanogaster*
**DmMalA5**	AAF59085	*Drosophila melanogaster*
**DmMalA6**	AAS64893	*Drosophila melanogaster*
**DmMalA7**	AAF59084	*Drosophila melanogaster*
**DmMalA8**	AAF59083	*Drosophila melanogaster*
**DmMalB1**	AAF53127	*Drosophila melanogaster*
**DmMalB2**	AAF53128	*Drosophila melanogaster*
**AdAmy**	ETN67705	*Anopheles darlingi*
**AgAgm1**	AGAP012401	*Anopheles gambiae*
**AgAgm2**	AGAP012400	*Anopheles gambiae*
**BdMalA1**	XM_011207650	*Bactrocera dorsalis*
**CcMalA1**	XM_004534550	*Ceratitis capitata*
**CqAmy**	XM_001847477	*Culex quinquefasciatus*
**Cqglu**	XM_001851434	*Culex quinquefasciatus*
**MdMalA1**	XM_005185607	*Musca domestica*
**NlMal**	JN382244	*Nilaparvata lugens*
**AmHbg1**	AB253415	*Apis mellifera*
**AmHbg2**	AB253416	*Apis mellifera*
**AmHbg3**	D79208	*Apis mellifera*
**ApAPS1**	DQ223541	*Acyrthosiphon pisum*

Table S2. List of primers in in the RT-PCR analysis

Primer	Sequence (5'--3')	Purpose
**PxSUH1RT-F**	GCGAGCACAGAGTCTGAGTA	RT-PCR of *PxSUH1*
**PxSUH1RT-R**	GAGGTAGAACTGTTGACGAGCC	RT-PCR of *PxSUH1*
**PxSUH2RT-F**	TCTAACGCCCGAGACTTTGT	RT-PCR of *PxSUH2*
**PxSUH2RT-R**	CCTAACTCCTCGCCCTGGTA	RT-PCR of *PxSUH2*
**PxrplRT-F**	AAGAAGAGGACGAAACGG	RT-PCR of *rpl*
**PxrplRT-R**	GACCAGCACCTTACGGAAAC	RT-PCR of *rpl*
